# Affective computing in virtual reality: emotion recognition from brain and heartbeat dynamics using wearable sensors

**DOI:** 10.1038/s41598-018-32063-4

**Published:** 2018-09-12

**Authors:** Javier Marín-Morales, Juan Luis Higuera-Trujillo, Alberto Greco, Jaime Guixeres, Carmen Llinares, Enzo Pasquale Scilingo, Mariano Alcañiz, Gaetano Valenza

**Affiliations:** 1 Instituto de Investigación e Innovación en Bioingeniería, Universitat Politècnica de València, València, Spain; 2 Bioengineering and Robotics Research Centre E Piaggio & Department of Information Engineering, University of Pisa, Pisa, Italy

## Abstract

Affective Computing has emerged as an important field of study that aims to develop systems that can automatically recognize emotions. Up to the present, elicitation has been carried out with non-immersive stimuli. This study, on the other hand, aims to develop an emotion recognition system for affective states evoked through Immersive Virtual Environments. Four alternative virtual rooms were designed to elicit four possible arousal-valence combinations, as described in each quadrant of the Circumplex Model of Affects. An experiment involving the recording of the electroencephalography (EEG) and electrocardiography (ECG) of sixty participants was carried out. A set of features was extracted from these signals using various state-of-the-art metrics that quantify brain and cardiovascular linear and nonlinear dynamics, which were input into a Support Vector Machine classifier to predict the subject’s arousal and valence perception. The model’s accuracy was 75.00% along the arousal dimension and 71.21% along the valence dimension. Our findings validate the use of Immersive Virtual Environments to elicit and automatically recognize different emotional states from neural and cardiac dynamics; this development could have novel applications in fields as diverse as Architecture, Health, Education and Videogames.

## Introduction

Affective Computing (AfC) has emerged as an important field of study in the development of systems that can automatically recognize, model and express emotions. Proposed by Rosalind Picard in 1997, it is an interdisciplinary field based on psychology, computer science and biomedical engineering^1^. Stimulated by the fact that emotions are involved in many background processes^2^ (such as perception, decision-making, creativity, memory, and social interaction), several studies have focused on searching for a reliable methodology to identify the emotional state of a subject by using machine learning algorithms.

Thus, AfC has emerged as an important research topic. It has been applied often in education, healthcare, marketing and entertainment^3–6^, but its potential is still under development. Architecture is a field where AfC has been infrequently applied, despite its obvious potential; the physical-environment has on a great impact, on a daily basis, on human emotional states in general^7^, and on well-being in particular^8^. AfC could contribute to improve building design to better satisfy human emotional demands^9^.

Irrespective of its application, Affective Computing involves both emotional classification and emotional elicitation. Regarding emotional classification, two approaches have commonly been proposed: discrete and dimensional models. On the one hand, the former posits the existence of a small set of basic emotions, on the basis that complex emotions result from a combination of these basic emotions. For example, Ekman proposed six basic emotions: anger, disgust, fear, joy, sadness and surprise^10^. Dimensional models, on the other hand, consider a multidimensional space where each dimension represents a fundamental property common to all emotions. For example, the “Circumplex Model of Affects” (CMA)^11^ uses a Cartesian system of axes, with two dimensions, proposed by Russell and Mehrabian^12^: valence, i.e., the degree to which an emotion is perceived as positive or negative; and arousal, i.e., how strongly the emotion is felt.

In order to classify emotions automatically, correlates from, e.g., voice, face, posture, text, neuroimaging, and physiological signals are widely used^13^. In particular, several computational methods are based on variables associated with central nervous system (CNS) and autonomic nervous system (ANS) dynamics^13^. On the one hand, the use of CNS is justified by the fact that human emotions originate in the cerebral cortex, involving several areas in their regulation and feeling. In this sense, the electroencephalogram (EEG) is one of the techniques most used to measure CNS responses^14^, also through the use of wearable devices. On the other hand, a wider class of affective computing studies consider ANS changes elicited by specific emotional states. In this sense, experimental results over the last three decades show that Heart Rate Variability (HRV) analyses can provide unique and non-invasive assessments of autonomic functions on cardiovascular dynamics^15,16^. To this extent, there has been a great increase over the last decade in research and commercial interest in wearable systems for physiological monitoring. The key benefits of these systems are their small size, lightness, low-power consumption and, of course, their wearability^17^. The state of the art^18–20^ on wearable systems for physiological monitoring highlight that: i) surveys predict that the demand for wearable devices will increase in the near future; ii) there will be a need for more multimodal fusion of physiological signals in the near future; and iii) machine learning algorithms can be merged with traditional approaches. Moreover, recent studies present promising results on the development of emotion recognition systems through using wearable sensors instead of classic lab sensors, through HRV^21^ and EEG^22^.

Regarding emotional elicitation, the ability to reliably and ethically elicit affective states in the laboratory is a critical challenge in the process of the development of systems that can detect, interpret and adapt to human affect^23^. Many methods of eliciting emotions have been developed to evoke emotional responses. Based on the nature of the stimuli, two types of method are distinguished, the active and the passive. Active methods can involve behavioural manipulation^24^, social psychological methods with social interaction^25^ and dyadic interaction^26^. On the other hand, passive methods usually present images, sounds or films. With respect to images, one of the most prominent databases is the International Affective Picture System (IAPS), which includes over a thousand depictions of people, objects and events, standardized on the basis of valence and arousal^23^. The IAPS has been used in many studies as an elicitation tool in emotion recognition methodologies^15^. With respect to sound, the most used database is the International Affective Digitalised Sound System (IADS)^27^. Some researchers also use music or narrative to elicit emotions^28^. Finally, audio-visual stimuli, such as films, are also used to induce different levels of valence and arousal^29^.

Even when, as far we know, elicitation has been carried out with a non-immersive stimulus, it has been shown that these passive methods have significant limitations due to the importance of immersion for eliciting emotions through the simulation of real experiences^30^. In the present, Virtual Reality (VR) represents a novel and powerful tool for behavioural research in psychological assessment. It provides simulated experiences that create the sensation of being in the real world^31,32^. Thus, VR makes it possible to simulate and evaluate spatial environments under controlled laboratory conditions^32,33^, allowing the isolation and modification of variables in a cost and time effective manner, something which is unfeasible in real space^34^. During the last two decades VR has usually been displayed using desktop PCs or semi-immersive systems such as CAVEs or Powerwalls^35^. Today, the use of head-mounted displays (HMD) is increasing: these provide fully-immersive systems that isolate the user from external world stimuli. These provide a high degree of immersion, evoking a greater sense of presence, understood as the perceptual illusion of non-mediation and a sense of “being-there”^36^. Moreover, the ability of VR to induce emotions has been analysed in studies which demonstrate that virtual environments do evoke emotions in the user^34^. Other works confirm that Immersive Virtual Environments (IVE) can be used as emotional induction tools to create states of relaxation or anxiety^37^, basic emotions^38,39^, and to study the influence of the users cultural and technological background on emotional responses in VR^40^. In addition, some works show that emotional content increases sense of presence in an IVE^41^ and that, faced with the same content, self-reported intensity of emotion is significantly greater in immersive than in non-immersive environments^42^. Thus, IVEs, showing 360° panoramas or 3D scenarios through a HMD^43^, are powerful tools for psychological research^43^,.

Taking advantage of the IVE’s potentialities, in recent years some studies have used IVE and physiological responses, such as EEG, HRV and EDA, in different fields. Phobias^44–47^, disorders^48^, driving and orientation^49,50^, videogames^51^, quality of experience^52^, presence^53^ and visualization technologies^54^, are some examples of these applications. Particularly in emotion research, arousal and relaxation have been analysed in outdoor^55,56^ and indoor^57^ IVEs using EDA. Therefore, the state of the art presents the following limitations: (1) few studies analyse physiological responses in IVEs and, in particular, using an affective approach; (2) there are few validated emotional IVE sets which include stimuli with different levels of arousal and valence: and, (3) there is no affective computing research that tries to automatically recognize the user’s mood in an IVE through physiological signals and machine learning algorithms.

In this study, we propose a new AfC methodology capable of recognizing the emotional state of a subject in an IVE in terms of valence and arousal. Regarding stimuli, IVEs were designed to evoke different emotional states from an architectural point of view, by changing physical features such as illumination, colour and geometry. They were presented through a portable HMD. Regarding emotion recognition, a binary classifier will be presented, which uses effective features extracted from EEG and HRV data gathered from wearable sensors, and combined through nonlinear Support Vector Machine (SVM)^15^ algorithms.

## Material and Methods

### Experimental context

This work is part of a larger research project that attempts to characterize the use of VR as an affective elicitation method and, consequently, develop emotion recognition systems that can be applied to 3D or real environments.

**Figure 1:**
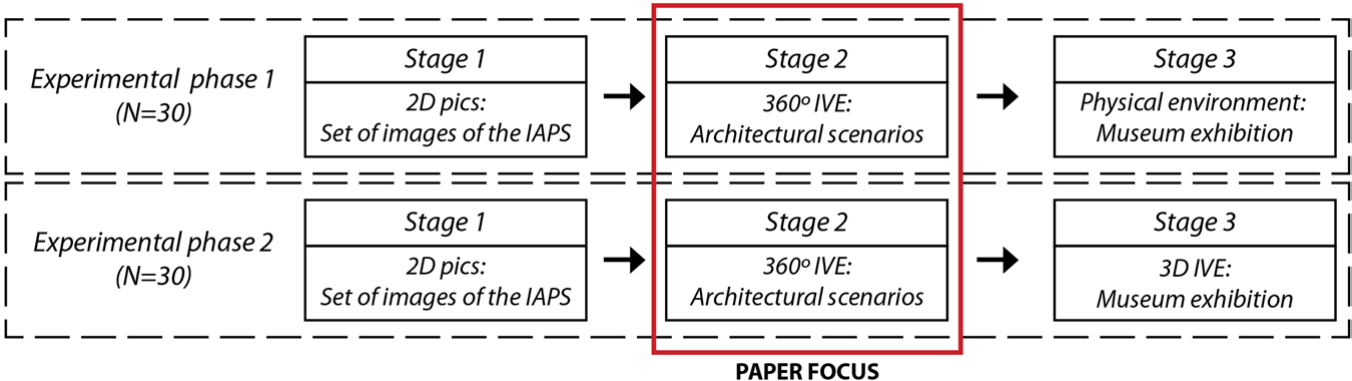
Experimental phases of the research.

An experimental protocol was designed to acquire the physiological responses of subjects in 4 different stimuli presentation cases: 2D desktop pictures, a 360° panorama IVE, a 3D scenario IVE and a physical environment. The experiment was conducted in two distinct phases that presented some differences. Both phases were divided into 3 stages; the results of the experiment are at Fig. 1. Between each stage, signal acquisition was temporarily halted and the subjects rested for 3 minutes on a chair. Stage 1 consisted of emotion elicitation through a desktop PC displaying 110 IAPS pictures, using a methodology detailed in previous research^15^. Stage 2 consisted of emotion elicitation using an HMD based on a new IVE set with four 360° panoramas. Finally, stage 3 consisted of the free exploration of a museum exhibition.

In the present paper we focus on an analysis of stage 2. The experimental protocol was approved by the ethics committee of the Polytechnic University of Valencia and informed consent was obtained from all participants. All methods and experimental protocols were performed in accordance with the guidelines and regulations of the local ethics committee of the Polytechnic University of Valencia.

### Participants

A group of 60 healthy volunteers, suffering neither from cardiovascular nor evident mental pathologies, was recruited to participate in the experiment. They were balanced in terms of age (28.9 ± 5.44) and gender (40% male, 60% female). Inclusion criteria were as follows: age between 20 and 40 years; Spanish nationality; having no formal education in art or fine art; having no previous experience of virtual reality; and not having previously visited the particular art exhibition. They were divided into 30 subjects for the first phase and 30 for the second.

To ensure that the subjects constituted a homogeneous group, and that they were in a healthy mental state, they were screened by i) the Patient Health Questionnaire (PHQ-9)^58^ and ii) the Self-Assessment Manikin (SAM)^59^.

PHQ-9 is a standard psychometric test used to quantify levels of depression^58^. Significant levels of depression would have affected the emotional responses. Only participants with a score lower than 5 were included in the study. The test was presented in the Spanish language as the subjects were native Spanish speakers. SAM tests were used to detect if any subject had an emotional response that could be considered as an outlier, with respect to a standard elicitation, in terms of valence and arousal. A set of 8 IAPS pictures^60^ (see Table 1), representative of different degrees of arousal and valence perception, was scored by each subject after stage 1 of the experiment. The z-score of each subject’s arousal and valence score was calculated using the mean and deviation of the IAPS’s published scores^60^. Subjects that had one or more z-scores outside of the range −2.58 and 2.58 (α = 0.005) were excluded from further analyses. Therefore, we retained subjects whose emotional responses, caused by positive and negative pictures, in different degrees of arousal, belonged to 99% of the IAPS population. In addition, we rejected subjects if their signals presented issues, e.g., disconnection of the sensors during the elicitation or if artefacts affected the signals. Taking these exclusions into account, the number of valid subjects was 38 (age: 28.42 ± 4.99; gender: 39% male, 61% female).

**Table 1:** Arousal and valence score of selected IAPS pictures from^56^.

IAPS picture	Arousal	Valence
7234	3.41 ± 2.29	4.01 ± 1.32
5201	3.20 ± 2.50	7.76 ± 1.44
9290	4.75 ± 2.20	2.71 ± 1.35
1463	4.61 ± 2.56	8.17 ± 1.48
9181	6.20 ± 2.23	1.84 ± 1.25
8380	5.84 ± 2.34	7.88 ± 1.37
3102	6.92 ± 2.50	1.29 ± 0.79
4652	7.24 ± 2.09	7.68 ± 1.64

### Set of Physiological Signals and Instrumentation

**Figure 2:**
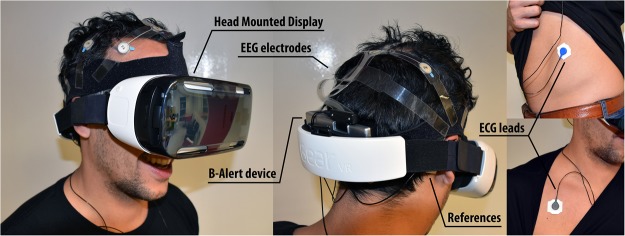
Exemplary experimental set-up.

The physiological signals were acquired using the B-Alert x10 (Advanced Brain Monitoring, Inc., USA) (Fig. 2). It provides an integrated approach for wireless wearable acquisition and recording of electroencephalographic (EEG) and electrocardiographic (ECG) signals, sampled at 256 Hz. EEG sensors were located in the frontal (Fz, F3 and F4), central (Cz, C3 and C4) and parietal (POz, P3, and P4) regions with electrode placements on the subjects’ scalps based on the international 10–20 electrode placement. A pair of electrodes placed below the mastoid was used as reference, and a test was performed to check the conductivity of the electrodes, aiming to keep the electrode impedance below 20kΩ. The left ECG lead was located on the lowest rib and the right lead on the right collarbone.

### Stimulus elicitation

We developed an affective elicitation system by using architectural environments displayed by 360° panoramas implemented in a portable HMD (Samsung Gear VR). This combination of environments and display-format was selected due to its capacity for evoking affective states. The bidirectional influence between the architectural environment and the user’s affective-behavioural response is widely accepted: even subtle variations in the space may generate different neurophysiological responses^61^. Furthermore, the 360° panorama-format provided by HMD devices is a valid set-up to evoke psychological and physiological responses similar to those evoked by physical environments^54^. Thus, following the combination of the arousal and valence dimensions, which gives the four possibilities described in the CMA^62^, four architectural environments were proposed as representative of four emotional states.

The four architectural environments were designed based on Kazuyo Sejima’s “Villa in the forest” scenario^63^. This architectural work was considered by the research team as an appropriate base from which to make the modifications designed to generate the different affective states.

The four base-scenario configurations were based on different modifications of the parameters of three design variables: illumination, colour, and geometry. Regarding illumination, the parameters “colour temperature”, “intensity”, and “position” were modified. The modification of the “colour temperature” was based on the fact that higher temperature may increase arousal, being registrable at the neurophysiological level^64,65^. “Intensity” was also modified in the same way to try to increase or reduce arousal. The “position” of the light was direct, in order to try to increase arousal, and indirect to reduce it. The modifications of these last two parameters were based on the design experience of the research team. Regarding colour, the parameters “tone”, “value”, and “saturation” were modified. The modification of these parameters was performed jointly on the basis that warm colours increase arousal and cold ones reduce it, being registrable at the psychological^66^ and neurophysiological levels^67–71^. Regarding geometry, the parameters “curvature”, “complexity”, and “order” were modified. “Curvature” was modified on the basis that curved spaces generate a more positive valence than angular, being registrable at psychological and neurophysiological levels^72^. The modification of the parameters “complexity” and “order” was performed jointly. This was based on three conditions registrable at the neurophysiological level: (1) high levels of geometric “complexity” may increase arousal and low levels may reduce arousal^73^; (2) high levels of “complexity” may generate a positive valence if they are submitted to “order”, and negative valence if presented disorderly^74^; and (3) content levels of arousal generated by geometry may generate a more positive valence^75^. The four architectural environments were designed on this basis. Table 2 shows the configuration guidelines chosen to elicit the four affective states.

**Table 2:** Configuration guidelines chosen in each architectural environment configuration.

		High-Arousal & Negative-Valence (Room 1)	High-Arousal & Positive-Valence (Room 2)	Low-Arousal & Negative-Valence (Room 3)	Low-Arousal & Positive-Valence (Room 4)
Illumination	Colour temperature	7500 K	7500 K	3500 K	3500 K
	Intensity	High	High	Low	Low
	Position	Mainly Direct	Mainly Direct	Mainly Indirect	Mainly Indirect
Colour	Tone	Warm colours	Warm colours	Cold colours	Cold colours
	Value				
	Saturation				
Geometry	Curvature	Rectilinear	Curved	Rectilinear	Curved
	Complexity	High	Low-Medium	Medium-High	Low
	Order	Low	High	Low-Medium	High

In a technical sense, the four architectural environments were developed in similar ways. The process consisted of modelling and rendering. Modelling was performed by using Rhinoceros v5.0 (www.rhino3d.com). The 3D-models used for the four architectural environments were 3446946, 3490491, 3487660, and 3487687 polygons. On completion of this process, they were exported in.dwg format for later rendering. The rendering was performed using the VRay engine v3.00.08 (www.vray.com), operating with Autodesk 3ds Max v2015 (www.autodesk.es). 15 textures were used for each of the four architectural environments. Configured as 360° panoramas, renders were exported in.jpg format with resolutions of 6000 × 3000 pixels at 300 dots per inch. These were implemented in the Samsung Gear VR HMD device. This device has a stereoscopic screen of 1280 × 1440 pixels per eye and a 96° field of view, supported by a Samsung Note 4 mobile telephone with a 2.7 GHz quad-core processor and 3GB of RAM. The reproduction of the architectural 360° panoramas was fluid and uninterrupted.

**Figure 3:**
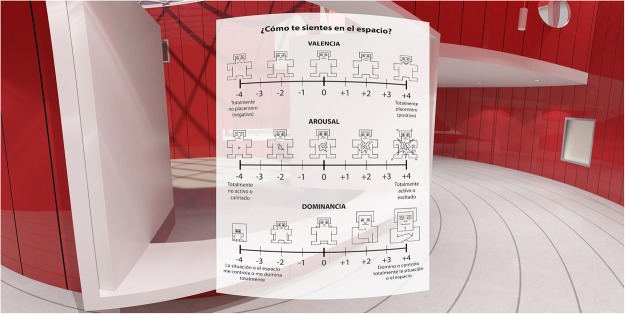
Example of SAM questionnaire embedded in the room 1. Simulation developed using Rhinoceros v5.0, VRay engine v3.00.08 and Autodesk 3ds Max v2015.

**Figure 4:**
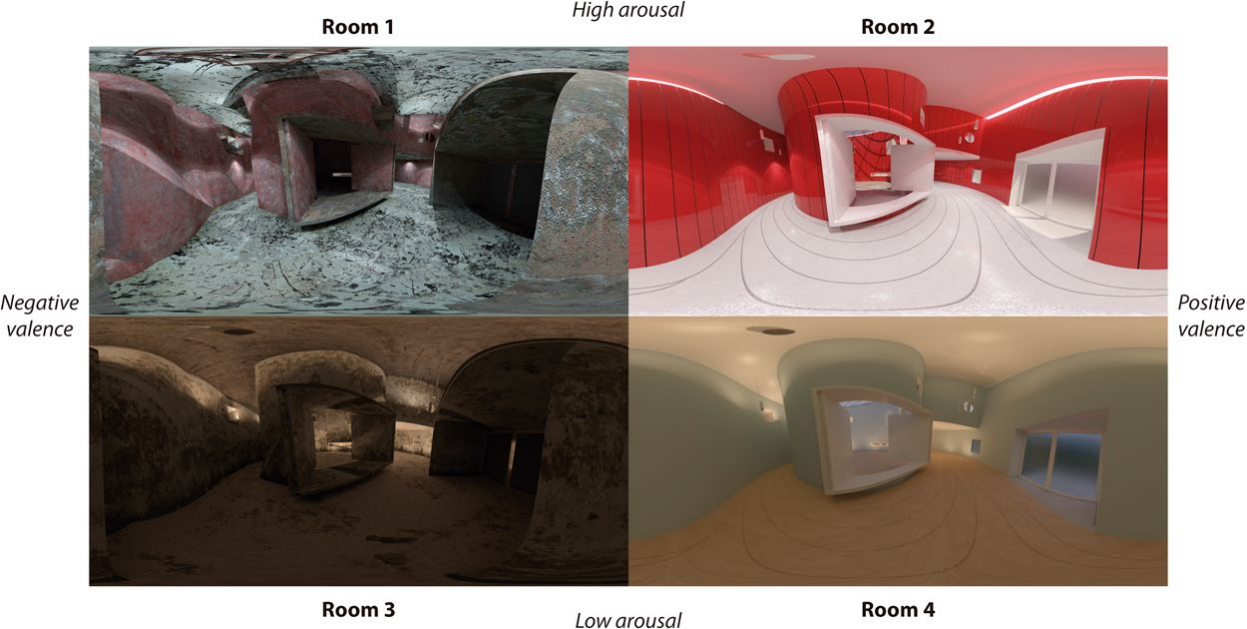
360° panoramas of the four IVEs. Simulations developed using Rhinoceros v5.0, VRay engine v3.00.08 and Autodesk 3ds Max v2015.

Prior to the execution of the experimental protocol, a pre-test was performed in order to ensure that the architectural 360° panoramas would elicit the affective states for which they had been designed. It was a three-phased test: individual questionnaires, a focus-group session conducted with some respondents to the questionnaire and individual validation-questionnaires. The questionnaires asked the participants to evaluate the architectural 360° panoramas. A SAM questionnaire, embedded in the 360° panorama, was used, with evaluations ranging from −4 (totally disagree) to 4 (totally agree) for all the emotion dimensions. 15 participants (8 men and 7 women) completed the questionnaires. First, the participants freely viewed each architectural environment, then the SAM questionnaires were presented and the answers given orally. Figure 3 shows an example of one of these questionnaires. After the questionnaire sessions had been completed, a focus group session, which was a carefully managed group discussion, was conducted^76^. Five of the participants (3 men and 2 women) with the most unfavourable evaluations in phase 1 were selected as participants and one of the members of the research team, with previous focus-group experience, moderated. The majority of the changes were performed to Room 3, due to the discordances between the self-assessment and their theoretical quadrant. Once the changes were implemented, a similar evaluation to phase 1 was performed. Table 3 shows the arousal and valence ratings of the four architectural 360° panoramas of this pre-test phase. After these phases, no new variations were considered necessary. This procedure allowed us to assume some initial reliability in the design of the architectural environments. Figure 4 shows these final configurations. High quality images of the stimuli are included in the supplementary material.

**Table 3:** Arousal and Valence resulted in the pre-test with 15 participants. The scores are averaged using mean and standard deviation for a Likert scale between −4 to +4.

	Arousal	Valence
High-Arousal & Negative-Valence (Room 1)	2.23 ± 1.59	−2.08 ± 1.71
High-Arousal & Positive-Valence (Room 2)	1.25 ± 1.33	1.31 ± 1.38
Low-Arousal & Negative-Valence (Room 3)	−0.69 ± 1.65	−1.46 ± 1.33
Low-Arousal & Positive-Valence (Room 4)	−2.31 ± 1.30	1.92 ± 1.50

None of the pre-test participants was included in the main study. Regarding the experimental protocol, each room was presented for 1.5 minutes and the sequence of presentation was counter-balanced using the Latin Square method. After viewing the rooms, the users were asking to orally evaluate the emotional impact of each room using a SAM questionnaire embedded in the 360° photo.

### Signal processing

#### Heart rate variability

The ECG signals were processed to derive HRV series^77^. The artefacts were cleaned by the threshold base artefacts correction algorithm included in the Kubios software^78^. In order to extract the RR series, the well-known algorithm developed by Pan-Tompkins was used to detect the R-peaks^79^. The individual trends components were removed using the smoothness prior detrending method^80^.

We carried out the analysis of the standard HRV parameters, which are defined in the time and frequency domains, as well as HRV measures quantifying heartbeat nonlinear and complex dynamics^77^. All features are listed in Table 4.

**Table 4:** List of used HRV features.

Time domain	Frequency domain	Other
Mean RR	VLF peak	Pointcaré SD1
Std RR	LF peak	Pointcaré SD2
RMSSD	HF peak	Approximate Entropy (ApEn)
pNN50	VLF power	Sample Entropy (SampEn)
RR triangular index	VLF power %	DFA α1
TINN	LF power	DFA α2
	LF power %	Correlation dimension (D2)
	LF power n.u.	
	HF power	
	HF power %	
	HF power n.u.	
	LF/HF power	
	Total power	

Time domain features include average (Mean RR) and standard deviation (Std RR) of the RR intervals, the root mean square of successive differences of intervals (RMSSD), and the ratio between the number of successive RR pairs having a difference of less than 50 ms and the total number of heartbeat analyses (pNN50). The triangular index was calculated as a triangular interpolation of the HRV histogram. Finally, TINN is the baseline width of the RR histogram, evaluated through triangular interpolation.

In order to obtain the frequency domain features, a power spectrum density (PSD) estimate was calculated for the RR interval series by a Fast Fourier Transform based on Welch’s periodogram method. The analysis was carried out in three bands: very low frequency (VLF, <0.04 Hz), low frequency (LF, 0.04–0.15 Hz) and high frequency (HF, 0.12–0.4 Hz). For each frequency band, the peak value was calculated, corresponding to the frequency with the maximum magnitude. The power of each frequency band was calculated in absolute and percentage terms. Moreover, for the LF and HF bands, the normalized power (n.u.) was calculated as the percentage of the signals subtracting the VLF to the total power. The LF/HF ratio was calculated in order to quantify sympatho-vagal balance and to reflect sympathetic modulations^77^. In addition, the total power was calculated.

Regarding the HRV nonlinear analysis, many measures were extracted, as they are important quantifiers of cardiovascular control dynamics mediated by the ANS in affective computing^15,16,77,81^. Pointcaré plot analysis is a quantitative-visual technique, whereby the shape of a plot is categorized into functional classes. The plot provides summary information as well as detailed beat-to-beat information on heart behaviour. SD1 is related to the fast beat-to-beat variability in the data, whereas SD2 describes the longer-term variability of R–R^77^. Approximate Entropy (ApEn) and Sample Entropy (SampEn) are two entropy measures of HRV. ApEn detects the changes in underlying episodic behaviour not reflected in peak occurrences or amplitudes^82^, whereas SampEn statistics provide an improved evaluation of time-series regularity and provide a useful tool in studies of the dynamics of human cardiovascular physiology^83^. DFA correlations are divided into short-term and long-term fluctuations through the α1 and α2 features. Whereas α1 represents the fluctuation in the range of 4–16 samples, α2 refers to the range of 16–64 samples^84^. Finally, the correlation dimension is another method for measuring the complexity or strangeness of the time series; it is explained by the D2 feature. It is expected to give information on the minimum number of dynamic variables needed to model the underlying system^85^.

#### Electroencephalographic signals

**Figure 5:**
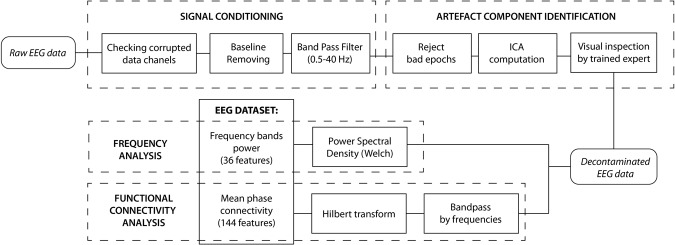
Block scheme of the EEG signal processing steps.

In order to process the EEG signals, the open source toolbox EEGLAB^86^ was used. The complete processing scheme is shown at Fig. 5.

Firstly, data from each electrode were analysed in order to identify corrupted channels. These were identified by computing the fourth standardized moment (kurtosis) along the signal of each electrode^87^. In addition, if the signal was flatter than 10% of the total duration of the experiment, the channel was classified as corrupted. If one of the nine channels was considered as corrupted, it could be interpolated from neighbouring electrodes. If more than one channel was corrupted, the subject would be rejected. Only one channel among all of the subjects was interpolated.

The baseline of EEG traces was removed by mean subtraction and a band pass filter between 0.5 and 40 Hz was applied. The signal was divided into epochs of one second and the intra-channel kurtosis level of each epoch was computed in order to reject the epochs highly damaged by noise^87^. In addition, automatic artefact detection was applied, which rejects the epoch when more than 2 channels have samples exceeding an absolute threshold of >100.00 µV and a gradient of 70.00 µV between samples^88^.

The Independent Component Analysis (ICA)^89^ was then carried out using infomax algorithm to detect and remove components due to eye movements, blinks and muscular artefacts. Nine source signals were obtained (one per electrode). A trained expert manually analysed all the components, rejecting those related to artefacts. The subjects who had more than 33% of their signals affected by artefacts were rejected.

After the pre-processing, spectral and functional connectivity analyses were performed.

EEG spectral analysis, using Welch’s method^90^, was performed to estimate the power spectra in each epoch, with 50% overlapping, within the classical frequency bandwidth θ (4–8 Hz), α (8–12 Hz), β (13–25 Hz), γ (25–40 Hz). Frequency band δ (less than 4 Hz) was not taken into account in this study because it relates to deeper stages of sleep. In total, 36 features were obtained from the nine channels and 4 bands.

A functional connectivity analysis was performed using Mean Phase Coherence^91^, for each pair of channels:

1 $${R}^{2}=E{[\cos ({\rm{\Delta }}\varphi )]}^{2}+E{[sin({\rm{\Delta }}\varphi )]}^{2}$$

where $R$ is the MPC, Δ*ϕ* represents the relative phase diference between two channels derived from the instantaneous difference of the analytics signals from the Hilbert transform, and $E$ is the expectation operator. By definition, MPC values ranged between 0 and 1. In the case of strong phase synchronization between two channels, the MPC is close to 1. If the two channels are not synchronized, the MPC remains low. 36 features were derived from each possible combination of a pair of 9 channels in one specific band. In total, 144 features were created using the 4 bands analysed.

### Feature reduction and machine learning

Each room was presented for 1.5 minutes and was considered as an independent stimulus. In order to characterize each room, all HRV features were calculated using this time window. In the case of EEG, in both the frequency band power and mean phase connectivity analyses, we considered the mean of all the epochs of each stimulus as the representative value of the stimulus time window. Altogether, 209 features described each stimulus for each subject. Due to the high-dimensional feature space obtained, a feature reduction strategy was adopted for decreasing this dimension. We implemented the well-known Principal Component Analysis method (PCA)^92^. This mathematical method is based on the linear transformation of the different variables in the principal components, which can be assembled in clusters. We select the features that explain 95% of the variability of the dataset. The PCA was applied three times: (1) in the HRV set, reducing the features from 29 to 3; (2) in the frequency band power analysis of the EEG, reducing the features from 36 to 4; and (3) in the mean phase coherency analysis of the EEG, reducing the features from 144 to 12. Hence, the feature reduction strategy reduces our features to a total of 19.

The machine learning strategy could be summarized as follows:

1. To divide the dataset into training and test sets.
2. The development of the model (parameter tuning and feature selection) using cross-validation in the training set.
3. To validate the model using the test set.

Firstly, the dataset was sliced randomly into 15% for the test set (5 subjects) and 85% for the training set (33 subjects). In order to calibrate the model, the Leave-One-Subject-Out (LOSO) cross-validation procedure was applied to the training set using Support Vector Machine (SVM)-based pattern recognition^93^. Within the LOSO scheme, the training set was normalized by subtracting the median value and dividing this by the median absolute deviation over each dimension. In each of the 36 iterations, the validation set consisted of one specific subject and he/she was normalized using the median and deviation of the training set.

**Figure 6:**
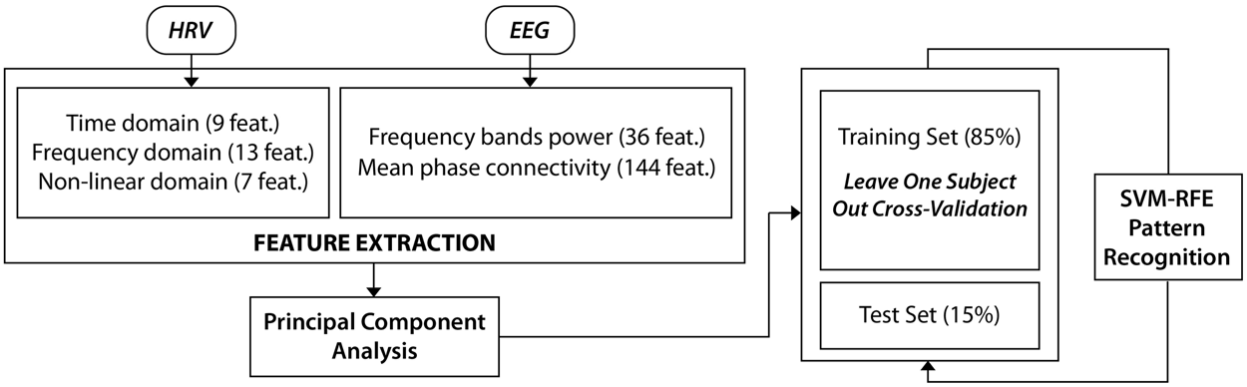
Overview of the feature reduction and classification chain.

Regarding the algorithm, we used a C-SVM optimized using a sigmoid kernel function, changing the parameters of cost and gamma using a vector with 15 parameters logarithmically spaced between 0.1 and 1000. Additionally, in order to explore the relative importance of all the features in the classification problem we used a support vector machine recursive feature elimination (SVM-RFE) procedure in a wrapper approach (RFE was performed on the training set of each fold and we computed the median rank for each feature over all folds). We specifically chose a recently developed, nonlinear SVM-RFE, which includes a correlation bias reduction strategy in the feature elimination procedure^94^. After the cross-validation, using the parameters and feature set obtained, the model was applied to the test set that had not previously been used. The self-assessment of each subject was used as the output of the arousal and valence model. The evaluation was bipolarized in positive/high (>0) and negative/low (<=0). All the algorithms were implemented by using Matlab© R2016a, endowed with an additional toolbox for pattern recognition, i.e., LIBSVM^95^. A general overview of the analysis is shown in Fig. 6.

## Results

### Subjects’ self-assessment

**Figure 7:**
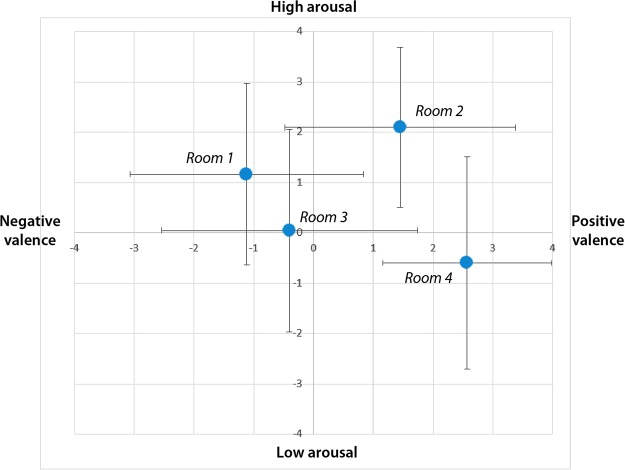
Self-assessment score in the IVEs using SAM and a Likert scale between −4 and +4. Blue dots represent the mean whereas horizontal and vertical lines represent standard deviation.

Figure 7 shows the self-assessment of the subjects for each IVE averaged using mean and standard deviation in terms of arousal (Room 1: 1.17 ± 1.81, Room 2: 2.10 ± 1.59, Room 3: 0.05 ± 2.01, Room 4: −0.60 ± 2.11) and valence (Room 1: −1.12 ± 1.95, Room 2: 1.45 ± 1.93, Room 3: −0.40 ± 2.14, Room 4: 2.57 ± 1.42). The representation follows the CMA space. All rooms are located in the theoretical emotion quadrant for which they were designed, except for Room 3 that evokes more arousal than hypothesized. Due to the non-Gaussianity of data (p < 0.05 from the Shapiro-Wilk test with null hypothesis of having a Gaussian sample), Wilcoxon signed-rank tests were applied. Table 5 presents the result of multiple comparisons using Tukey’s Honestly Significant Difference Procedure. Significant differences were found in the valence dimension between the negative-valence rooms (1 and 3) and the positive-valence rooms (2 and 4). Significant differences were found in the arousal dimension between the high-arousal rooms (1 and 2) and the low-arousal rooms (3 and 4), but not for pairs 1 and 3. Therefore, the IVEs statistically achieve all the desired self-assessments except for arousal perception in Room 3, which is higher than we hypothesized. After the bipolarization of scores (positive/high >0), they are balanced (61.36% high arousal and 56.06% positive valence).

**Table 5:** Signification test of the self-assessment of the emotional rooms.

IVE		p-value	
		Arousal	Valence
1	2	0.052	10–6 (***)
1	3	0.195	0.152
1	4	0.007 (**)	10–9 (***)
2	3	10–5 (***)	0.015 (*)
2	4	10–8 (***)	0.068
3	4	0.606	10–7 (***)

### Arousal classification

**Figure 8:**
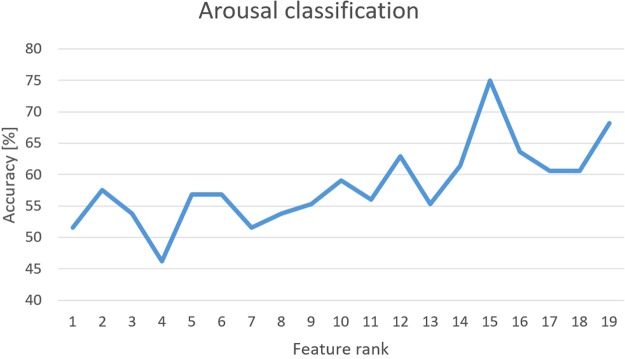
Recognition accuracy of arousal in cross-validation as a function of the feature rank estimated through the SVM-RFE procedure.

Table 6 shows the confusion matrix of cross validation and the total average accuracy (75.00%), distinguishing two levels of arousal using the first 15 features selected by the nonlinear SVM-RFE algorithm. The F-Score of arousal classification is 0.75. The changes in accuracy depending on number of features are shown in Fig. 8, and Table 7 presents the list of features used. Table 8 shows the confusion matrix of the test set and the total average accuracy (70.00%) using the parameters and the feature set defined in the cross-validation phase. The F-score of arousal classification is 0.72 in the test set.

**Table 6:** Confusion matrix of cross-validation using SVM classifier for arousal level. Values are expressed as percentages. Total Accuracy: 75.00%.

Arousal	High	Low
High	82.72	17.28
Low	37.25	62.75

**Table 7:** Selected features ordered by their median rank over every fold computed during the LOSO procedure for arousal classification.

Rank	Feature
1	EEG MPC PCA 8
2	EEG MPC PCA 9
3	EEG MPC PCA 11
4	EEG MPC PCA 10
5	EEG MPC PCA 7
6	EEG MPC PCA 12
7	EEG Band Power PCA 3
8	EEG Band Power PCA 1
9	HRV PCA 1
10	EEG Band Power PCA 4
11	EEG Band Power PCA 2
12	HRV PCA 3
13	EEG MPC PCA 4
14	HRV PCA 2
15	EEG MPC PCA 5

**Table 8:** Confusion matrix of test set using SVM classifier for arousal level. Values are expressed as percentages. Total Accuracy: 70.00%.

Arousal	High	Low
High	75.00	25.00
Low	33.33	66.67

### Valence classification

**Figure 9:**
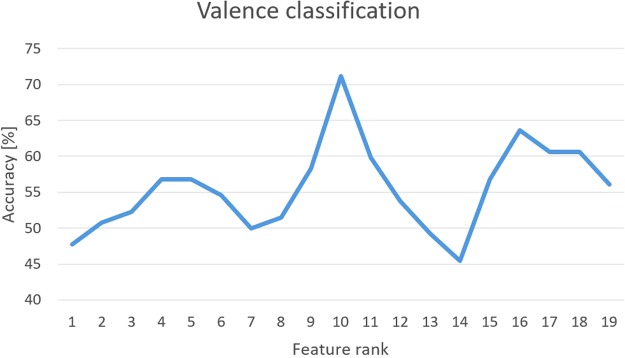
Recognition accuracy of valence in cross-validation as a function of the feature rank estimated through the SVM-RFE procedure.

Table 9 shows the confusion matrix of the cross validation and total average accuracy (71.21%), distinguishing two levels of valence using the first 10 features selected by the nonlinear SVM-RFE algorithm. The F-Score of the valence classification is 0.71. The changes in accuracy depending on the number of features are shown in Fig. 9, and Table 10 presents the list of features used. Table 11 shows the confusion matrix of the test set and total average accuracy (70.00%), using the parameters and the feature set defined in the cross-validation phase. The F-score of the valence classification was 0.70 in the test set.

**Table 9:** Confusion matrix of cross-validation using SVM classifier for valence level. Values are expressed as percentages. Total Accuracy: 71.21%.

Valence	Positive	Negative
Positive	71.62	28.38
Negative	29.31	70.69

**Table 10:** Selected features ordered by their median rank over every fold computed during the LOSO procedure for valence classification.

Rank	Feature
1	EEG MPC PCA 8
2	EEG MPC PCA 6
3	EEG MPC PCA 11
4	EEG MPC PCA 7
5	EEG MPC PCA 10
6	EEG MPC PCA 12
7	EEG MPC PCA 9
8	EEG Band Power PCA 3
9	EEG Band Power PCA 4
10	EEG MPC PCA 2

**Table 11:** Confusion matrix of test set using SVM classifier for valence level. Values are expressed as percentages. Total Accuracy: 70.00%.

Valence	Positive	Negative
Positive	75.00	25.00
Negative	37.50	62.50

## Discussion

The purpose of this study is to develop an emotion recognition system able to automatically discern affective states evoked through an IVE. This is part of a larger research project that seeks to analyse the use of VR as an affective elicitation method, in order to develop emotion recognition systems that can be applied to 3D or real environments. The results can be discussed on four levels: (1) the ability of IVEs to evoke emotions; (2) the ability of IVEs to evoke the same emotions as real environments; (3) the developed emotion recognition model; and (4), the findings and applications of the methodology.

Regarding the ability of the IVEs to evoke emotions, four versions of the same basic room design were used to elicit the four main arousal-valence combinations related to the CMA. This was achieved by changing different architectural parameters, such as illumination, colour and geometry. As shown in Fig. 7 and Table 5, proper elicitation was achieved for Room 1 (high arousal and negative valence), Room 2 (high arousal and positive valence) and Room 4 (low arousal and positive valence), but it overlapped somewhat with the arousal-valence representation in Room 3: despite the satisfactory pre-test, in the event it evoked higher arousal and valence than expected. This is due to the difficulties we experienced in designing a room to evoke negative emotion with low arousal. It should be noted that IAPS developers may also have experienced this problem because only 18.75% of the pics are situated in this quadrant^60^. Other works based on processing valence and arousal using words show that a U-model exists in which arousal increases in agreement with valence intensity regardless of whether it is positive or negative^96^. Hence, for future works, Room 3 will be redesigned to decrease its arousal and valence and a self-assessment with a larger sample will be performed, by questionnaire, to robustly assess the IVE. Nonetheless, after thresholding the individual self-assessment scores to discern 2 classes (high/low), the IVE set was balanced in arousal and valence. Therefore, we could conclude that the proposed room set can satisfactorily evoke the four emotions represented by each quadrant of the CMA.

To this extent, although previous studies have presented IVEs capable of evoking emotional states in a controlled way^97^, to the best of our knowledge we have presented the first IVE suite capable of evoking a variety of levels of arousal and valence based on CMA. Moreover, the suite was tested through a low-cost portable HMD, the Samsung Gear, therefore increasing the possible applications of the methodology. High quality images of the stimuli are included in the supplementary material. This represents a new tool that can contribute in the field of psychology, in general, and in the affective computing field, in particular, fostering the development of novel immersive affective elicitation using IVEs.

There are still some topics that need to be researched, relating to the capacity of the IVE display formats, to ensure that they evoke the same emotions as real environments. Studies comparing display formats show that the 360° IVEs offer results closer to reality, according to the participants’ psychological responses, and 3D IVEs do so according to their physiological responses^54^. Moreover, it is quite possible that IVEs will offer the best solutions at both psychological and physiological levels as they become even more realistic, providing a real improvement not only at the visual and auditory levels but also at the haptic^98^. In addition, 3D IVEs allow users to navigate and interact with the environment. Hence, there are reasons to think that they could be powerful tools for developing applications for affective computing, but studies comparing human responses in real and simulated IVE are scarce^99–101^, especially regarding emotional responses; these studies are required. Moreover, every year the resolution of Head Mounted Displays is upgraded, which brings them closer to eye resolution. Thus, it is possible that in some years the advances in Virtual Reality hardware will make the present methodology more powerful. In addition, works comparing VR devices with different levels of immersion are needed in order to give researchers the best set-ups to achieve their aims. In future works, we need to consider all these topics to improve the methodology.

Regarding the emotion recognition system, we present the first study that develops an emotion recognition system using a set of IVEs as a stimulus elicitation and proper analyses of physiological dynamics. The accuracy of the model was 75.00% along the arousal dimension and 71.21% along the valence dimension in the phase of cross-validation, with average of 70.00% along both dimensions in the test set. They all present a balanced confusion matrix. The accuracies are considerably higher than the chance level, which is 58% in brain signal classification and statistical assessment (n = 152, 2-classes, p = 0.05)^102^. Although the accuracy is lower than other studies of emotion recognition in images^15^ and sounds^27^, our results present a first proof of concept that suggests that it is possible to recognize the emotion of a subject elicited through an IVE. The research was developed with a sample of 60 subjects, who were carefully screened to demonstrate agreement with a “standard” population reported in the literature^46^. It should be noted that the possible overfitting of the model was controlled using: (1) a feature reduction strategy with a PCA; (2) a feature selection strategy using a SVM-RFE; (3) a first validation of the model using LOSO cross-validation; and (4) a test validation using 5 randomly chosen subjects (15%), who had not been used before to train or perform the cross-validation of the model. In the arousal model, features derived from three-signal analyses were selected: 3/3 of HRV, 4/4 of EEG BandPower and 8/12 of EEG MPC. However, in the valence model only the EEG analysis was used: 0/3 of HRV, 2/4 of EEG BandPower and 8/12 EEG MPC. Moreover, in both models, the first six features selected by RFE-SVM were derived from an EEG MPC analysis. This suggests that cortical functional connectivity provides effective correlates of emotions in an IVE. Furthermore, according to recent evidence^22,103^, the reliability of emotion recognition outside of the laboratory environment is improved by wearables. In future experiments, these results could be optimized using further, maybe multivariate signal analyses and alternative machine learning algorithms^87^. In addition, the design of new, controlled IVEs that can increase the number of stimuli per subject, using more combinations of architectural parameters (colour, illumination and geometry), should also improve the accuracy and robustness of the model. In future studies, we will improve the set of stimuli presented including new IVEs in order to develop a large set of validate IVE stimuli to be used in emotion research.

The findings presented here mark a new step in the field of affective computing and its applications. Firstly, the methodology involved in itself a novel trial to overcome the limitations of passive methods of affective elicitation, in order to recreate more realistic stimuli using 360° IVEs. Nevertheless, the long-term objective is to develop a robust pre-calibrate model that could be applied in two ways: (1) in 3D environments that would allow the study of emotional responses to “real” situations in a laboratory environment through VR simulation using HMD devices and (2) in physical spaces. We hypothesize in both cases that the emotion recognition models developed through controlled 360° IVEs will work better than the models calibrated by non-immersive stimuli, such as IAPS. This approach will be discussed in future studies using stage 3 of the experimental protocol.

Regarding the implications for architecture, the methodology could be applied in two main contexts, research and commercial. On the one hand, researchers could analyse and measure the impact of different design parameters on the emotional responses of potential users. This is especially important due to the impossibility of developing researches in real or laboratory environments (e.g. analysing arousal changes caused by the pavement width on a street). The synergy of affective computing and virtual reality allows us to isolate a parameter design and measure the emotional changes provoked by making changes to it, while keeping the rest of the environment identical. This could improve the knowledge of the emotional impact that might be made by different design parameters and, consequently, facilitate the development of better practices and relevant regulations. On the other hand, this methodology could help architects and engineers in their decision-making processes for the design of built environments before construction, aiding their evaluations and the selection of the options that might maximize the mood that they want to evoke: for example, positive valence in a hotel room or a park, low arousal in a schoolroom or in a hospital waiting room and high arousal in a shop or shopping centre. Nevertheless, these findings could be applied to any other field that needs to quantify the emotional effects of spatial stimuli displayed by Immersive Virtual Environments. Health, psychology, driving, videogames and education might all benefit from this methodology.

## Electronic supplementary material

Supplementary materials

Exemplary physiological data

## Data Availability

The datasets generated during and/or analysed during the current study are available from the corresponding authors on reasonable request.
