# Supplementary material for: Affective computing in virtual reality: emotion recognition from brain and heartbeat dynamics using wearable sensors
Source: Sci Rep. 2018 Sep 12;8:13657. doi: 10.1038/s41598-018-32063-4 (PMC6135750; doi:10.1038/s41598-018-32063-4)
Supplement: Supplementary file 1 — Supplementary materials [file 41598_2018_32063_MOESM1_ESM.docx]

**Affective computing in virtual reality: emotion recognition from brain and heartbeat dynamics using wearable sensors**

Javier Marín-Morales^1,*^, Juan Luis Higuera-Trujillo^1^, Alberto Greco^2^, Jaime Guixeres^1^, Carmen Llinares^1^, Enzo Pasquale Scilingo^2^, Mariano Alcañiz^1^ and Gaetano Valenza^2^

^1^ Instituto de Investigación e Innovación en Bioingeniería, Universitat Politècnica de València, València, Spain

^2^ Bioengineering and Robotics Research Centre E Piaggio & Department of Information Engineering, University of Pisa, Pisa, Italy

* Corresponding author: jamarmo@i3b.upv.es

**SUPPLEMENTARY MATERIALS**

**Emotional rooms set**

High quality images of the stimuli are available at <http://personales.upv.es/jamarmo/emotionalrooms/>.
